# Complete Heart Block Complicating Takotsubo Syndrome: Case Report and Literature Review

**DOI:** 10.1155/2020/7614836

**Published:** 2020-08-19

**Authors:** Michael J. McGee, William Yu, Joshua McCarthy, Malcolm Barlow, Rosemary Hackworthy

**Affiliations:** ^1^School of Medicine and Public Health, University of Newcastle, Newcastle, NSW, Australia; ^2^Cardiovascular Department, John Hunter Hospital, Newcastle, NSW, Australia; ^3^Hunter Medical Research Institute, Newcastle, NSW, Australia

## Abstract

An 81-year-old woman presents with shortness of breath resulting in a diagnosis of picornavirus and complete heart block. Troponin was elevated and there was concern about acute coronary syndrome. The final diagnosis after echocardiogram and coronary angiogram was Takotsubo syndrome in addition to the heart block which required pacemaker insertion.

## 1. History of Presentation

An 81-year-old woman presented to a peripheral emergency department with a two-week history of shortness of breath. She had been commenced on oral antibiotics for a lower respiratory tract infection in the community.

### 1.1. Past Medical History

The patient has a history of hypertension, dyslipidaemia, osteoporosis, anxiety, and gastrooesophageal reflux disease.

### 1.2. Medications at Admission

Irbesartan 300 mg daily, hydrochlorothiazide 12.5 mg daily, thyroxine 50 mcg daily, rosuvastatin 10 mg daily, sertraline 25 mg daily, aspirin 100 mg daily, metoprolol 12.5 mg twice daily were the medications for the patients.

### 1.3. Clinical Course

**Figure 1:**
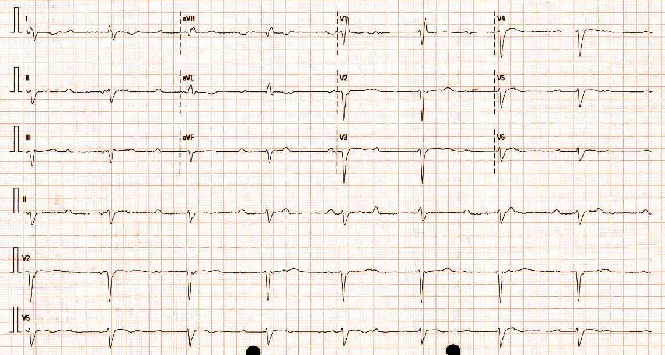
Electrocardiogram revealing complete heart block with a ventricular escape at approximately 48 beats per minute.

On assessment in the emergency department, the patient was found to be in complete heart block (CHB) with a ventricular escape of 48 bpm (Figure 1). She denied chest pain on presentation or recently. High sensitivity troponin was found to be elevated at 525 ng/L (<16). The patient was transported to the local tertiary hospital for the management of acute coronary syndrome and complete heart block. A respiratory viral PCR swab was sent prior to transport. The swab later returned positive for picornavirus. B-type natriuretic peptide (BNP) was elevated at 1174 ng/L (<266) and subsequent troponins fell.

**Figure 2:**
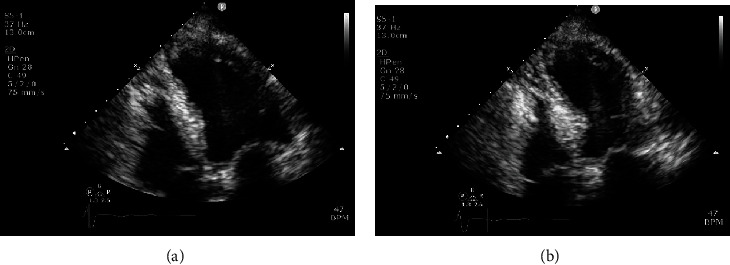
Transthoracic echocardiogram performed on presentation. Apical 4 chamber view, at end diastole (a) and end systolic (b) demonstrating left ventricular apical hypokinesis.

Echocardiogram after transfer revealed a large area of left ventricular apical hypokinesis, moderate atrial dilatation, and tricuspid regurgitation in the context of the known complete heart block (Figure 2 and supplementary figure 1).

**Figure 3:**
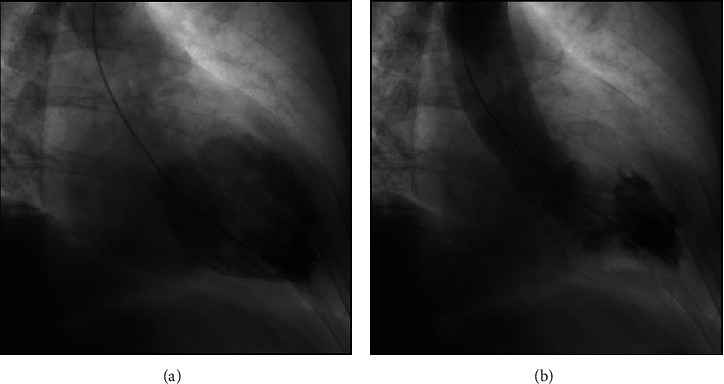
Cardiac catheterisation performed on day 9, left ventriculogram at end diastole (a) and end systole (b) demonstrating resolution of the apical hypokinesis.

Cardiac catheterisation was delayed secondary to inability to lie flat as a result of the respiratory infection, with cough fits and agitation without desaturation. This was performed on day 9 postpresentation. The coronary angiogram revealed nonobstructive coronary artery disease, with a focal 50% stenosis in the midleft circumflex coronary artery (supplemental figure 2). The left ventriculogram performed revealed near normalisation of the apical hypokinesis and an elevated end diastolic pressure of 20 mmHg (Figure 3). Laboratory findings are listed in Table 1.

### 1.4. Management

**Figure 4:**
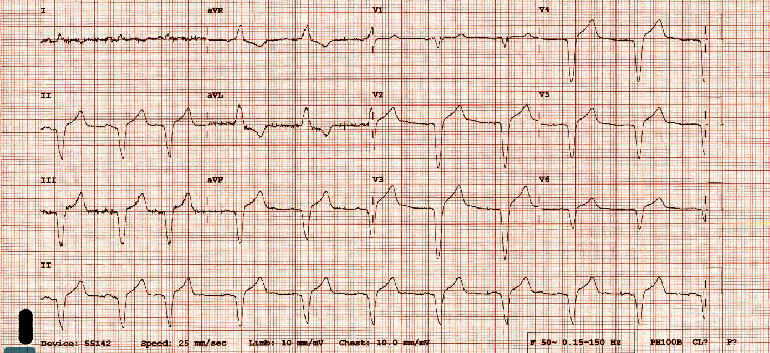
Electrocardiogram following implantation of a dual chamber pacemaker with atrial sensed, ventricular paced complexes.

The complete heart block continued after cessation of the metoprolol. The patient was initially managed conservatively as the ventricular escape was greater than 40 bpm without hypotension. The patient did have exacerbations of coughing and agitation when lying flat, which could be attributed to either heart failure or respiratory tract infection. Chest X-ray on presentation was not consistent with heart failure despite the raised BNP. The picornavirus infection was managed conservatively. Nine days after presentation, once the patient was able to lie flat without incident, a dual chamber Biotronik pacemaker was implanted, DDD 60–130 (Figure 4). The patient was discharged on aspirin, irbesartan, rosuvastatin, and sertraline.

### 1.5. Follow-Up

Interrogation of the pacemaker 6 weeks postimplantation revealed underlying complete heart block with 100% ventricular pacing, no tachyarrhythmias, and satisfactory device parameters. The patient remains independent in the community 6 months following discharge.

## 2. Discussion

The patient had previously been referred to a cardiologist as an outpatient due to concerns regarding cardiomegaly on chest X-ray and resistant hypertension. Investigations completed 6 months prior to the patient's hospitalisation revealed normal left ventricular size and systolic function on an echocardiogram and right bundle branch block on an electrocardiogram.

It appears that the Takotsubo syndrome (TTS) in our patient's case was precipitated by the lower respiratory tract infection with picornavirus. This phenomenon of an emotional or physiological insult as a trigger for TTS has been well described. This case is unusual given the presence of complete heart block in association with TTS at time of presentation. It is unclear when the patient developed complete heart block as the ventricular escape was robust and was well tolerated hemodynamically. There was preexisting right bundle branch block, and the possibility of progressive conduction disease is high.

The alternative diagnoses for this case include acute coronary syndrome and viral-induced myocarditis. Picornavirus family has been associated with viral myocarditis in other cases and series [1]. Cardiac magnetic resonance imaging could help distinguish in cases were the diagnosis is unclear. We felt the characteristics of the echocardiogram and angiogram were consistent with TTS, and subsequently, cardiac magnetic resonance imaging and cardiac biopsy were not pursued.

Acute coronary syndrome was another possible diagnosis, with spontaneous recanalization. We felt this was unlikely given that the extensive wall motion abnormality was beyond a single coronary artery territory (the patient did not have a left anterior descending that wrapped around the apex) and was disproportionate to the peak troponin.

Several case reports of TTS and CHB exist [2–4]. The clinical challenges are twofold: firstly, determining which precipitates the other, and secondly, determining the need for a cardiac implantable electronic device (CIED). If the TTS is secondary to CHB, then pacing will be required; however, if the CHB is secondary to TTS, then there is the possibility of recovery and a CIED can be avoided. Cases of both scenarios have been reported in the literature [5, 6].

CIED implantation is not a benign procedure with recent research documenting a high complication rate of approximately 10% [7]. There are case reports of TTS occurring post CIED implantation [8]. Case reports of TTS and CHB have described implantation of dual chamber pacemakers, biventricular pacemakers, and defibrillator for the treatment of CHB [9–11]. One of the largest consecutive series of TTS reported a CHB prevalence of 2.2% [6]. Including our case, we were able to identify 24 reported cases of CHB complicating TTS. In four of the cases, a CIED was not implanted due to the resolution of the heart block. In further two cases, the AV block resolved postimplantation of the CIED (Table 2).

## 3. Conclusion

TTS is not a benign condition and presents a number of clinical challenges. An association with complete heart block is not uncommon and often requires CIED implantation.

Take-home messages and learning objectives are as follows:

- Takotsubo syndrome can result in a wide range of dangerous arrhythmias, both brady and tachycardic
- Takotsubo syndrome can complicate other cardiovascular disease entities
- Most, but not all patients who have complete heart block and Takotsubo syndrome require a pacemaker

## Figures and Tables

**Table 1:** 

Value	Initial	Peak	Reference range
Troponin I	525	525	<16 ng/L
TSH	2.84		0.40-5.00 mIU/L
Sodium	131	139	135–145 mmol/L
Potassium	4.4	5.5	3.5–5.2 mmol/L
Creatinine	82	121	45-90*μ*mol/L
Calcium (corrected)	2.56	2.71	2.10-2.60 mmol/L
BNP	1174		<266 ng/L
CRP	38	49	<5 mg/L
WCC	11.5	14.1	4.0-11.0 × 10^9^/L
Haemoglobin	123	131	115-165 g/L

**Table 2:** 

Case	Year	Author	Age	Sex	TTS and CHB	CIED	F/U period (months)	Resolved
1	2004	Saito et al. [12]	86	F	1	Yes	NR	Unknown
2	2006	Nef et al. [13]	58	M	1	Yes	3	Yes
3	2006	Lee et al. [14]	72	F	1	No	<1	Yes
4	2007	Nault et al. [2]	62	F	1	Yes	24	Yes
5	2008	Kurisu et al. [9]	78	M	1	Yes	<1	Unknown
6			87	F	1	Yes	NR	Unknown
7	2009	Inoue et al. [3]	82	F	1	Yes	3	Unknown
8	2009	Kodama et al. [15]	57	F	1	Yes	NR	Unknown
9			39	F	1	Yes	NR	Unknown
10	2011	Siry et al. [16]	70	F	1	Yes	NR	Unknown
11	2012	Benouda et al. [17]	81	F	1	Yes	NR	Unknown
12	2012	Shanmugasundar et al. [4]	72	F	1	Yes	NR	Unknown
13	2013	Chadha et al. [18]	61	F	1	No	12	Yes
14	2013	Wakiya et al. [19]	86	F	1	Yes	12	No
15	2013	Sugiura et al. [5]	63	F	1	No	NR	Unknown
16	2015	Stiermaier et al. [6]	NR	NR	1	Yes	NR	Unknown
17			NR	NR	1	Yes	NR	Unknown
18			NR	NR	1	Yes	NR	Unknown
19			NR	NR	1	No	NR	Unknown
20	2015	Korantzopoulos et al. [20]	71	F	1	Yes	6	No
21	2017	Inayat et al. [11]	59	F	1	Yes	6	No
22	2018	Afzal et al. [21]	62	F	1	Yes	6	No
23	2019	Sakul et al. [10]	73	F	1	Yes	20	No
24	2020	McGee	81	F	1	Yes	3	No

NR: not reported.

## Abbreviations

CIED: Cardiac implantable electronic device
CHB: Complete heart block (third-degree atrioventricular block)
TTS: Takotsubo syndrome.

## Supplementary Materials

Supplementary 1 Supplemental Figure 1: Transthoracic echocardiogram performed on presentation. Apical 2 chamber view, at end diastole (left) and end systolic (right) demonstrating left ventricular apical hypokinesis. Supplemental figure 2: Coronary angiography, with the left coronary artery (left) and the right coronary artery (right) demonstrating non-obstructive coronary artery disease.

Supplementary 2 Supplemental Videos: Video 1: Transthoracic echocardiogram performed on presentation. Apical 2 chamber view demonstrating apical hypokinesis with preserved basal function consistent with takotsubo syndrome. Video 2: Transthoracic echocardiogram performed on presentation. Apical 3 chamber view demonstrating apical hypokinesis with preserved basal function consistent with takotsubo syndrome. Video 3: Transthoracic echocardiogram performed on presentation. Apical 4 chamber view demonstrating apical hypokinesis with preserved basal function consistent with takotsubo syndrome. Video 4: Cardiac catheterisation performed on day 9, left ventriculogram demonstrating resolution of the apical hypokinesis.
