# Supplementary material for: Complete Heart Block Complicating Takotsubo Syndrome: Case Report and Literature Review
Source: Case Rep Cardiol. 2020 Aug 19;2020:7614836. doi: 10.1155/2020/7614836 (PMC7453273; doi:10.1155/2020/7614836)
Supplement: Supplementary 1 — Supplemental Figure 1: Transthoracic echocardiogram performed on presentation. Apical 2 chamber view, at end diastole (left) and end systolic (right) demonstrating left ventricular apical hypokinesis. Supplemental figure 2: Coronary angiography, with the left coronary artery (left) and the right coronary artery (right) demonstrating non-obstructive coronary artery disease. [file 7614836.f1.docx]

Supplemental Figures:

Supplemental Figure 1:


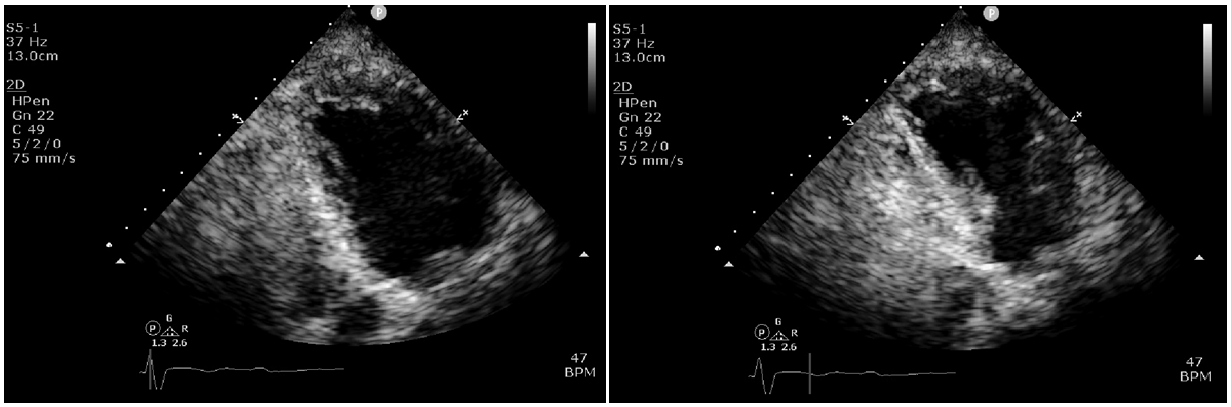


Transthoracic echocardiogram performed on presentation. Apical 2 chamber view, at end diastole (left) and end systolic (right) demonstrating left ventricular apical hypokinesis

Supplemental figure 2:


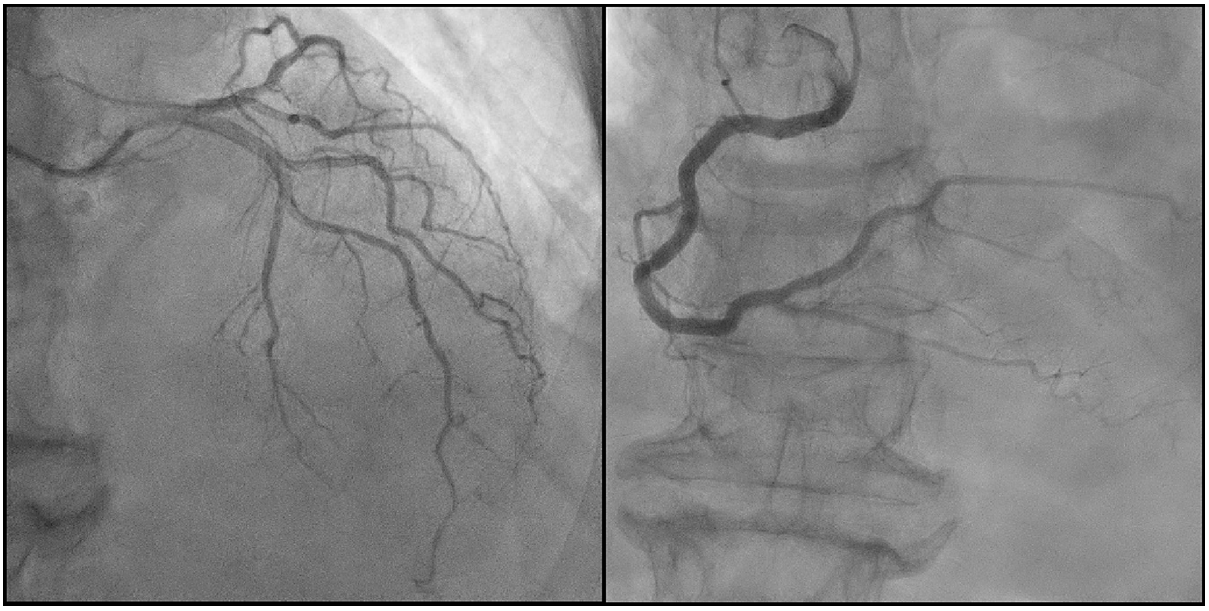


Coronary angiography, with the left coronary artery (left) and the right coronary artery (right) demonstrating non-obstructive coronary artery disease.
