# Supplementary figures and images for: Complete Heart Block Complicating Takotsubo Syndrome: Case Report and Literature Review
Source: Case Rep Cardiol. 2020 Aug 19;2020:7614836. doi: 10.1155/2020/7614836 (PMC7453273; doi:10.1155/2020/7614836)

## Slide 1
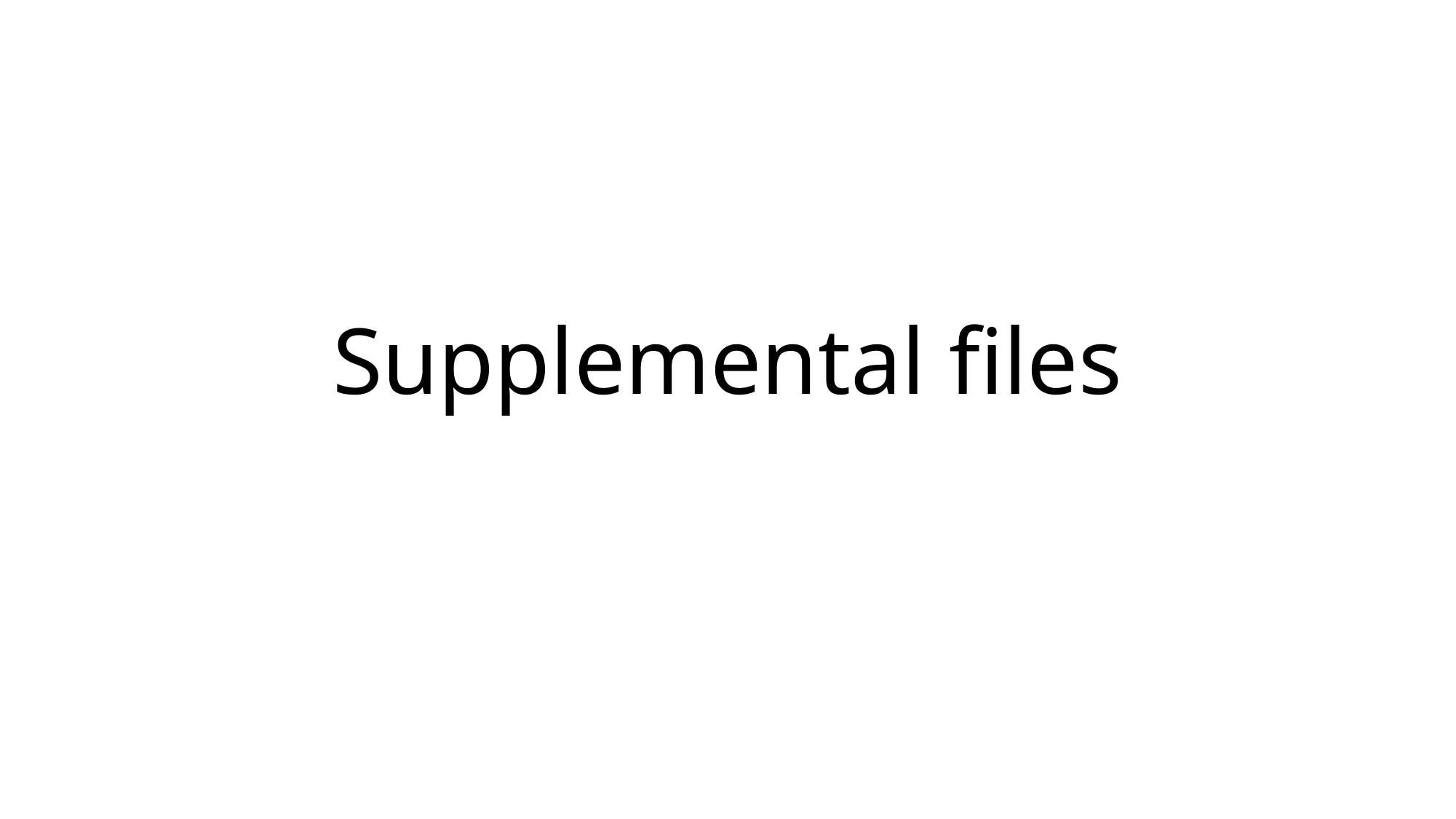

# Supplemental files

## Slide 2
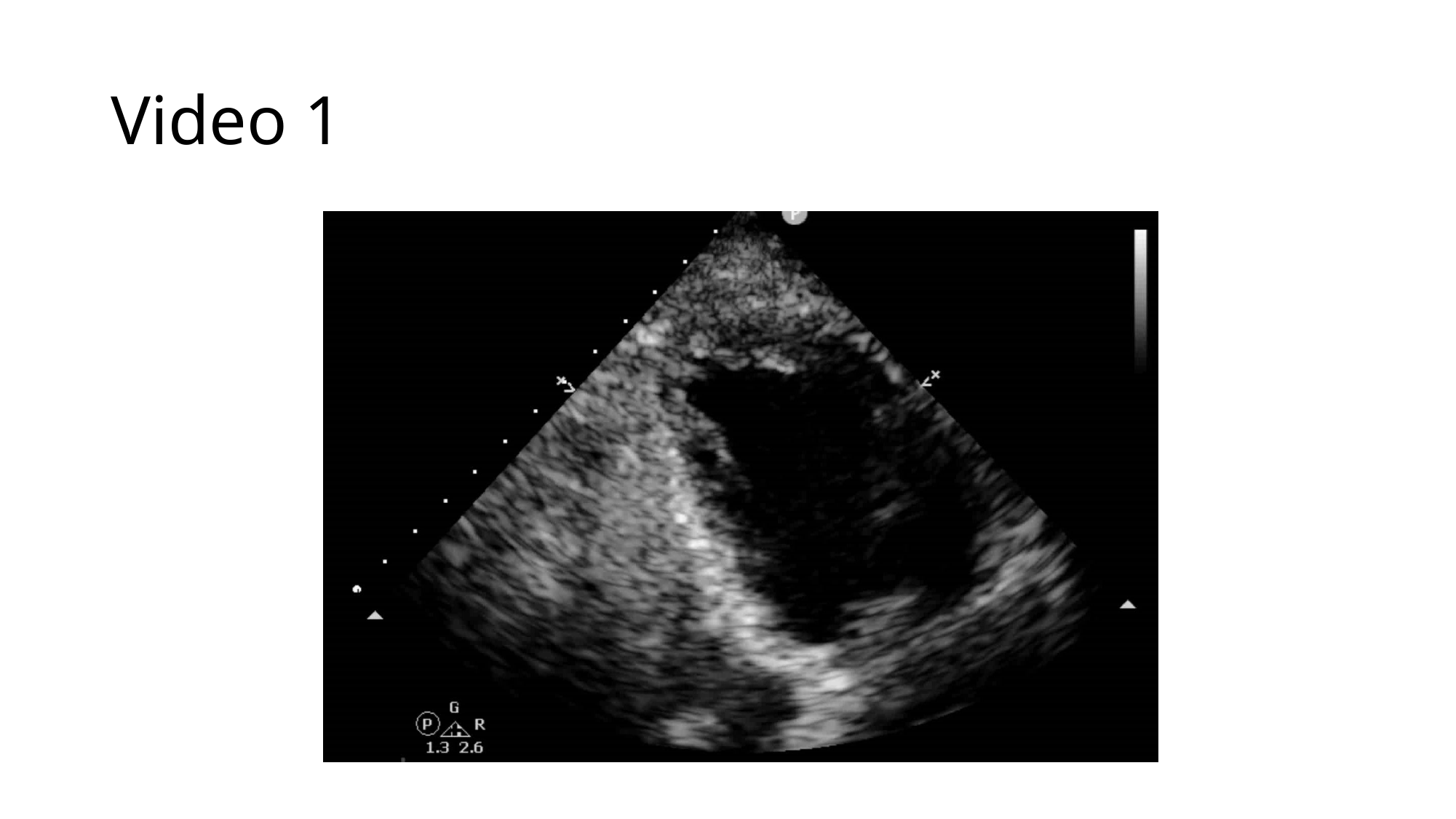

# Video 1

## Slide 3
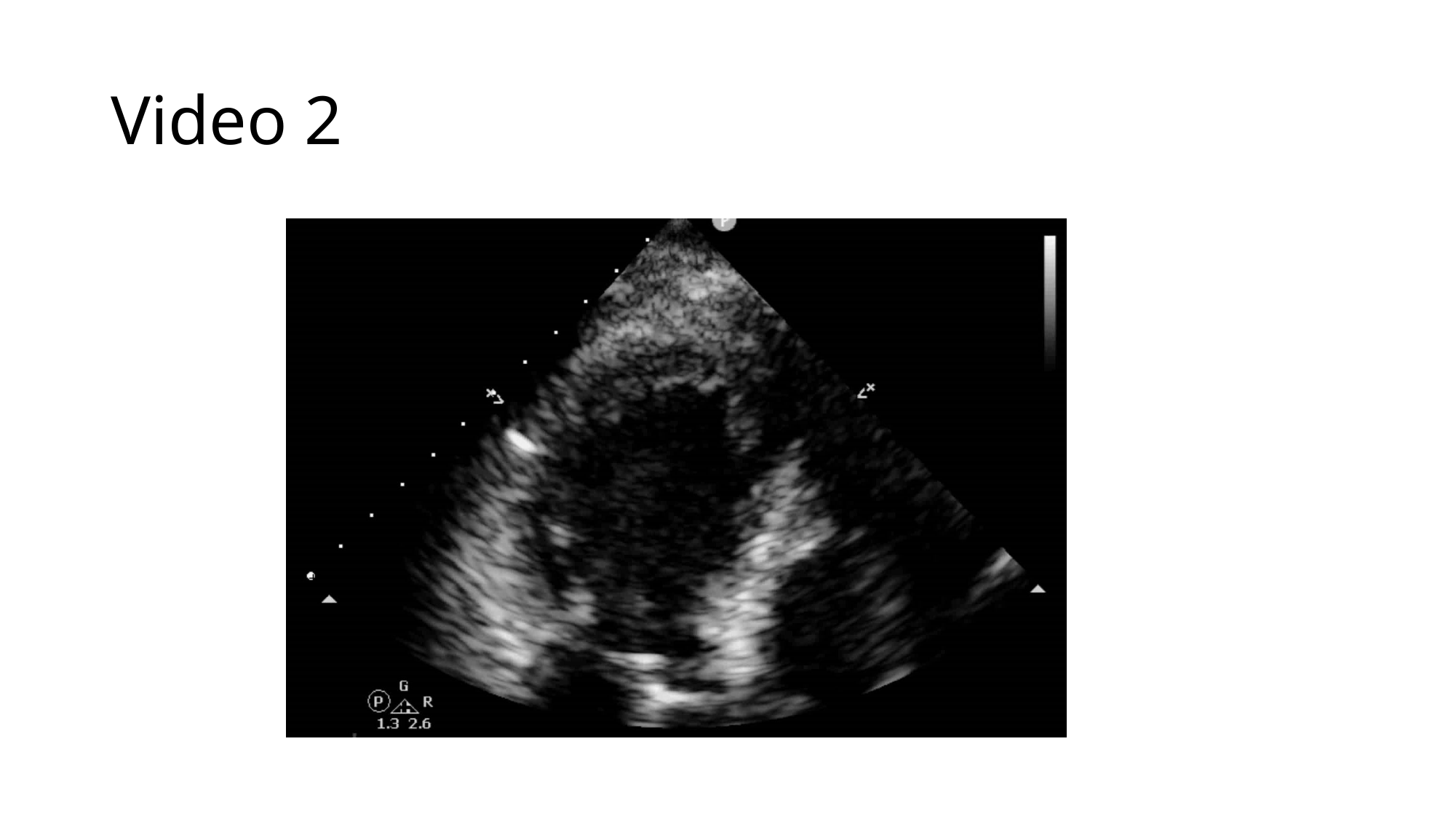

# Video 2

## Slide 4
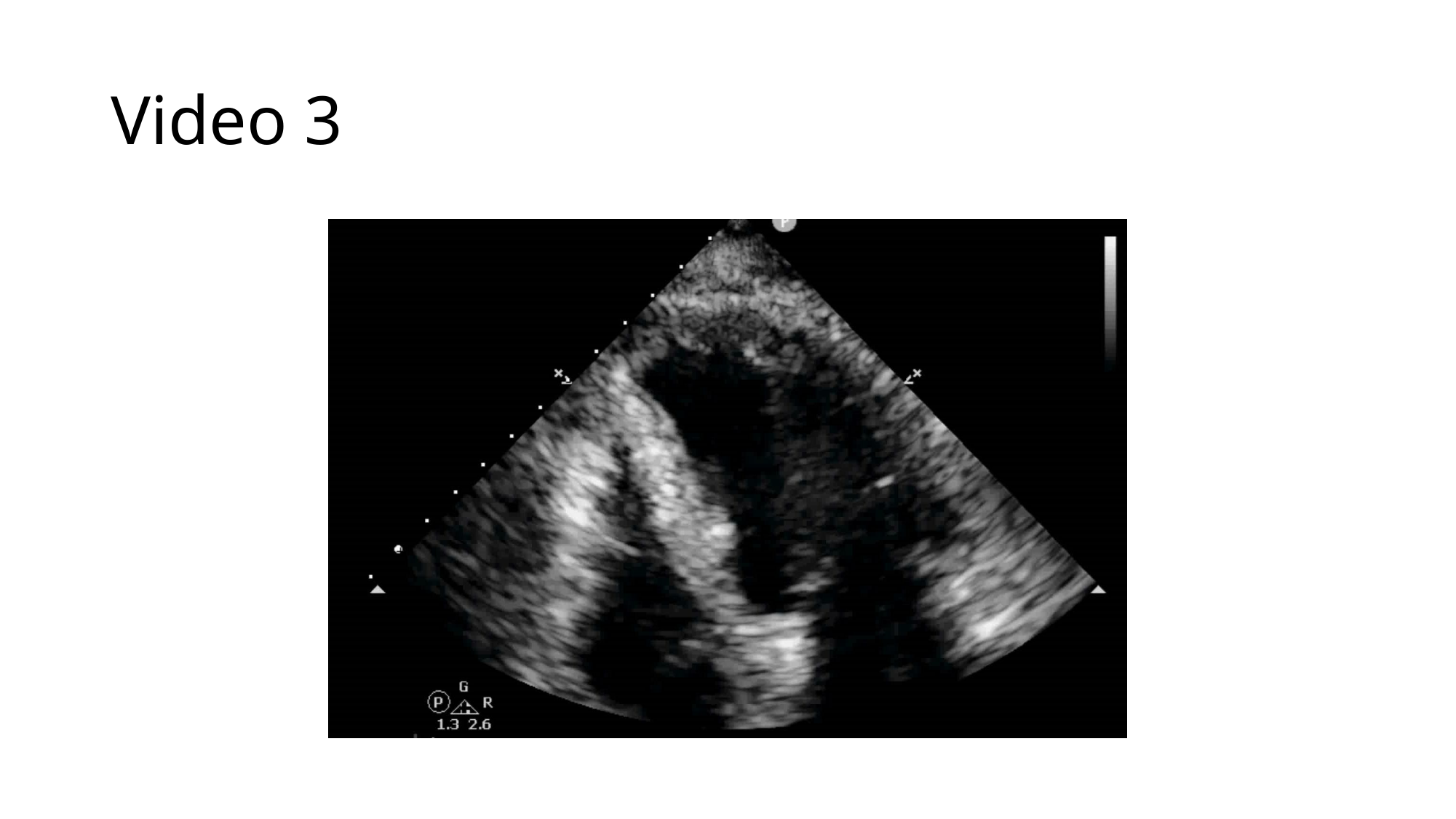

# Video 3

## Slide 5
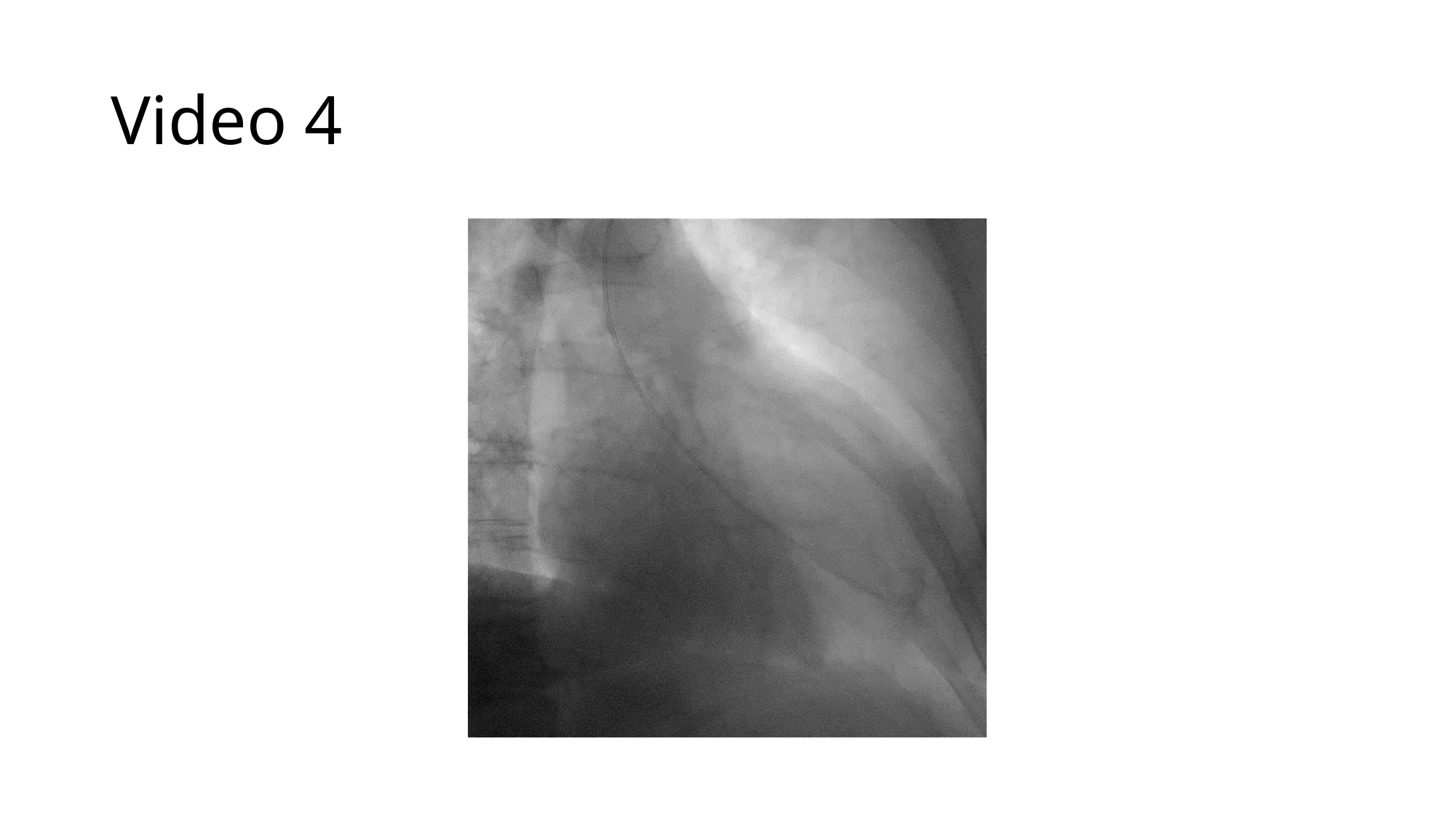

# Video 4

Supplement: Supplementary 2 — Supplemental Videos: Video 1: Transthoracic echocardiogram performed on presentation. Apical 2 chamber view demonstrating apical hypokinesis with preserved basal function consistent with takotsubo syndrome. Video 2: Transthoracic echocardiogram performed on presentation. Apical 3 chamber view demonstrating apical hypokinesis with preserved basal function consistent with takotsubo syndrome. Video 3: Transthoracic echocardiogram performed on presentation. Apical 4 chamber view demonstrating apical hypokinesis with preserved basal function consistent with takotsubo syndrome. Video 4: Cardiac catheterisation performed on day 9, left ventriculogram demonstrating resolution of the apical hypokinesis. [file 7614836.f2.pptx]
